# Cobalt Doping of Na_2_VTi(PO_4_)_3_ Enables a High-Energy NASICON-Type Cathode Material for Sodium-Ion Batteries

**DOI:** 10.3390/ma18112419

**Published:** 2025-05-22

**Authors:** Yu Zhang, Mengyao Wang, Hao Fan, Chenyang Huang, Mingfei Liu, Xiaofa Liang, Ping Hu, Xuanpeng Wang, Qin Wang, Fei Lv, Liang Zhou

**Affiliations:** 1 State Key Laboratory of Advanced Technology for Materials Synthesis and Processing, Wuhan University of Technology, Wuhan 430070, China; 281630@whut.edu.cn (Y.Z.); 281102@whut.edu.cn (M.W.); haofan8911@163.com (H.F.); 18438183410@163.com (C.H.); mingfeiliu359@gmail.com (M.L.); 344871@whut.edu.cn (X.L.); wxp122525691@whut.edu.cn (X.W.); 2 Key Laboratory of Intelligent Sensing System and Security of the Ministry of Education, Hubei Key Laboratory of Micro-Nanoelectronic Materials and Devices, School of Microelectronics, Hubei University, Wuhan 430062, China; 3 Zhongyu Feima New Material Technology Innovation Center (Zhengzhou) Co., Ltd., No. 60 Xuelan Road, High Technology Industrial Development Zone, Zhengzhou 450001, China; 4 Hubei Longzhong Laboratory, Wuhan University of Technology (Xiangyang Demonstration Zone), Xiangyang 441000, China; wangqinwr@hbwanrun.com (Q.W.); lvfei@hbwanrun.com (F.L.); 5 Hubei Wanrun New Energy Technology Co., Ltd., No. 557, Tianma Road, Yunyang Economic Development Zone, Shiyan 442003, China

**Keywords:** sodium-ion battery, cathode material, cobalt doping, NASICON structure, multi-electron redox reaction

## Abstract

Natrium superionic conductor (NASICON) compounds have emerged as a rising star in the field of sodium-ion batteries (SIBs) owing to their stable framework structure and high Na^+^ ionic conductivity. The NASICON-structured Na_2_VTi(PO_4_)_3_ manifests significant potential as Na^+^ storage material, characterized by decent rate capability and cyclability. However, the low redox potential of Ti^3+^/Ti^4+^ and undesirable energy density limit its practical applications. We developed a NASICON-structured Na_3_Co_2/3_V_2/3_Ti_2/3_(PO_4_)_3_ (NCTVP) cathode material by doping an appropriate amount of cobalt into Na_2_VTi(PO_4_)_3_. Cobalt doping introduces a Co^3+^/Co^2+^ redox couple at ~4.1 V and activates the V^5+^/V^4+^ redox at ~3.9 V, resulting in significantly increased medium discharge voltage and capacity. NCTVP demonstrates a high capacity of over 160 mAh g^−1^ at 20 mA g^−1^. With a medium discharge voltage of ~2.7 V, the energy density of NCTVP reaches 432.0 Wh kg^−1^. NCTVP also demonstrates desirable cycling stability (87.4% retention for 100 cycles at 50 mA g^−1^). In situ X-ray diffraction discloses a solid solution reaction mechanism for NCTVP, while the galvanostatic intermittent titration technique demonstrates fast Na^+^ diffusion kinetics. NCTVP also demonstrates high capacity and good cyclability in full cells. This contribution demonstrates an effective approach for the construction of NASICON materials for SIBs.

## 1. Introduction

The demand for renewable energy has grown significantly in recent years, making wind and solar power increasingly vital to the global energy landscape. However, environmental factors often restrict the practical use of renewable energy sources. This situation emphasizes the urgent need for energy storage systems (ESSs) [1,2,3,4,5,6]. Due to the affordability and plentiful availability of sodium, sodium-ion batteries (SIBs) have garnered considerable interest as a prospective solution for ESSs [7,8,9,10,11,12]. However, the relatively large ionic radius of sodium (1.06 Å) results in slow diffusion kinetics in electrode materials. Additionally, the relatively low structural stability of most SIB cathode materials further complicates the development of SIBs [13]. These challenges have hindered the commercialization of SIBs. To overcome these issues, the development of SIB cathode materials with high energy, high structural stability, and high safety has become a key focus of ongoing research.

Among the various SIB cathode materials, polyanionic compounds stand out due to their excellent structural stability and elevated redox potential. Natrium superionic conductors (NASICONs), a representative class of polyanionic compounds, show great potential in sodium storage due to their fast Na^+^ transport channels and high structural stability [14,15,16,17]. The general formula for NASICON-type phosphates is Na_x_M_y_(PO_4_)_3_ (M = V, Mn, Ti, Al, Co, and Zr), and their structure consists of “lantern-shaped” cells aligned along the c-axis, each comprising two [MO_6_] octahedra and three [PO_4_] tetrahedra. This arrangement gives ideal rate capability, structural stability, and cyclability to the material. Furthermore, the flexible atomic arrangement of NASICON materials provides opportunities for further modification and optimization.

Na_3_V_2_(PO_4_)_3_ (NVP) is a well-studied NASICON-type SIB cathode material. It is capable of carrying out reversible intercalation/de-intercalation with two Na^+^ ions, resulting in a theoretical capacity of 117 mAh g^−1^. In addition, NVP also manifests decent rate performance and cyclability [18]. However, the energy density and capacity of NVP are unsatisfactory, which limit its development. Additionally, vanadium (V) is not only expensive but also toxic, further hindering the wide practical application of NVP in SIBs [19].

To increase the energy density and reduce the toxicity of NVP, researchers have proposed various modification strategies. The main strategy involves partially or fully substituting V with alternative cations to promote multi-electron reactions and constructing carbon-based networks to improve electrical conductivity [20]. Manganese (Mn), due to its low cost and low toxicity, has emerged as an ideal candidate to substitute V. Na_4_MnV(PO_4_)_3_ (NMVP) with a NASICON structure material was first reported by Goodenough’s group [21]. Li et al. reported a series of Na_3+x_Mn_x_V_2−x_(PO_4_)_3_ materials with tunable V/Mn ratios [22]. Wang et al. introduced a Na_2_V_1−x_Ti_x_(PO_4_)_3_ material, which exhibited excellent rate performance and cyclability [23]. Hu et al. developed a high-performance Na_3.2_MnTi_0.8_V_0.2_(PO_4_)_3_ cathode material with an ideal energy density and discharge capacity [24]. However, the incorporation of Mn would decrease the stability and cycling performance of the NASICON-type compound, primarily due to the Jahn–Teller effect of Mn^3+^ [25]. Additionally, the Ti^3+^/Ti^4+^ redox couple shows a relatively low potential [26].

In this study, a Na_3_Co_2/3_Ti_2/3_V_2/3_(PO_4_)_3_/C/rGO material (NCTVP) is designed, where the introduction of Co effectively improves the capacity and redox potential. The well-designed NCTVP material demonstrates a capacity of over 160 mAh g^−1^ at 20 mA g^−1^ and good cyclability (87.4% retention over 100 cycles at 50 mA g^−1^). Furthermore, in situ X-ray diffraction (XRD) discloses a solid solution reaction mechanism for NCTVP. The NCTVP//commercial hard carbon (HC) full cell can also provide ideal capacity (over 130 mAh g^−1^) and cyclability (74.8% retention over 100 cycles at 50 mA g^−1^).

## 2. Materials and Methods

First, citric acid monohydrate (C_6_H_8_O_7_·H_2_O, 2.80 g, 13.3 mmol), sodium dihydrogen phosphate dihydrate (NaH_2_PO_4_·2H_2_O, 1.41 g, 9.0 mmol), ammonium metavanadate (NH_4_VO_3_, 0.234 g, 2.0 mmol), cobalt acetate tetrahydrate (Co(CH_3_COO)_2_·4H_2_O, 0.498 g, 2.0 mmol), anhydrous sodium citrate (C_6_H_5_O_7_Na_3_, 0.0687 g, 0.2 mmol), dihydroxybis (ammonium lactate), titanium aqueous solution ([CH_3_CH(O-)CO_2_NH_4_]_2_Ti(OH)_2_, 0.962 mL, 50 wt.%, 2.0 mmol), and reduced graphene oxide aqueous dispersion (5 mg mL^−1^, 30 mL) were dispersed in water, followed by continuous stirring for 3 h. These materials were bought from Aladdin Biochemical Technology Co. (Shanghai, China). The mixture was stirred at 80 °C for 8 h to remove the water. The obtained powder was pretreated at 350 °C for 5 h, followed by sintering at 650 °C for 8 h in high-purity nitrogen to obtain the Na_3_Co_2/3_Ti_2/3_V_2/3_(PO_4_)_3_/C/rGO (NCTVP) composite. The reduced graphene oxide was incorporated to enhance the electrical conductivity of NCTVP. For comparison, Na_2_TiV(PO_4_)_3_/C (NTVP) was also prepared using a similar method, with no Co(CH_3_COO)_2_·4H_2_O and more anhydrous sodium citrate (0.94 g, 6 mmol) added during synthesis.

## 3. Results and Discussion

**Figure 1:**
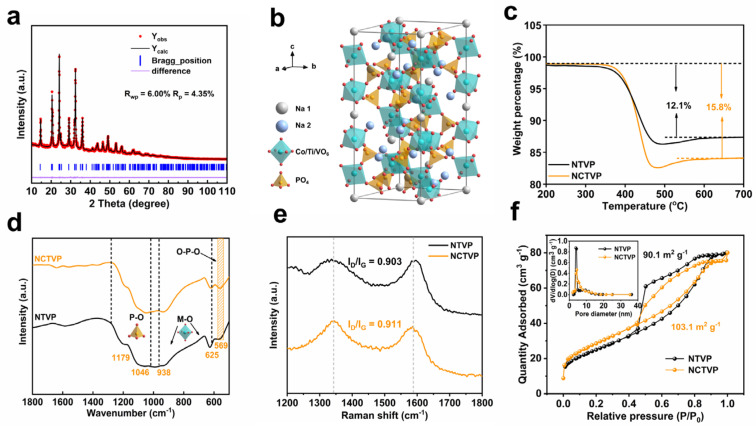
(**a**) XRD Rietveld refinement pattern of NCTVP; (**b**) schematic crystal structure of NCTVP; (**c**) TGA curves; (**d**) FT-IR spectra; (**e**) Raman spectra; (**f**) N_2_ adsorption–desorption isotherms and corresponding pore size distribution curves of NCTVP and NTVP.

The effect of Co^2+^ substitution on lattice parameters is studied using XRD Rietveld refinements (Figure 1a and Figure S1 and Tables S1 and S2). The refinement results indicate that both NCTVP and NTVP belong to the rhombohedral lattice structure with the R$\bar{3}$c space group. The cell parameters *a* and *b* of NCTVP (*a* = 8.7016 Å) are larger than those of NTVP (*a* = 8.6148 Å). However, its *c* value (*c* = 21.7675 Å) is smaller than that of NTVP (*c* = 21.8465 Å), which is caused by the reduced repulsive forces between adjacent MO_6_ octahedra. The cell volumes of NCTVP and NTVP are 1427.4 and 1404.1 Å^3^, respectively, indicating that the substitution of 33% of Ti^4+^ and V^3+^ by Co^2+^ with a larger ionic radius (0.72 Å for Co^2+^, 0.67 Å for V^3+^, and 0.61 Å for Ti^4+^) induces an expansion of the cell volume [27,28,29]. Figure 1b shows the crystal structure of NCTVP.

Thermogravimetric analysis (TGA) shows that the weight losses of the final calcined NCTVP and NTVP in the temperature range of 200 to 700 °C are 15.8 and 12.1 wt.%, respectively (Figure 1c). The weight losses are caused by the burn-off of carbon in air, which is formed by the decomposition of graphene oxide, citric acid, and organic species. Both cathode materials exhibit excellent thermal stability as no further weight loss occurs after the burn-off of carbon layers. Fourier transform infrared spectroscopy (FT-IR, Figure 1d) provides structural information on the samples. The peaks between 625 and 938 cm^−1^ are from the vibration of M–O bonds in the MO_6_ octahedra. The P–O bond presents a symmetric stretching signal at 1179 cm^−1^ [29], whereas asymmetric stretching is located at 1023 cm^−1^ [30]. The bending vibration of the O–P–O bond presents a band at 569 cm^−1^ [31]. These results further confirm the existence of phosphate groups in NCTVP and NTVP.

Raman spectra (Figure 1e) reveal the graphitization degree of carbon. The D band (1334 cm^−1^) and G band (1580 cm^−1^) are related to the disordered and graphitic nature of carbon, respectively. NCTVP and NTCP show I_D_/I_G_ ratios of 0.911 and 0.903, respectively. Low I_D_/I_G_ ratios indicate the partial graphitization of carbon, which is expected to enhance the conductivity and thus the electrochemical performance [32]. N_2_ sorption results (Figure 1f) provide information on the textural properties. The surface area of NCTVP (103.1 m^2^ g^−1^) is slightly higher than that of NTVP (90.1 m^2^ g^−1^) due to the higher carbon content attributed to the introduction of reduced graphene oxide. The pore size distribution (PSD) curves reveal that both NCTVP and NTVP have abundant porosity, which is beneficial for electrolyte infiltration.

**Figure 2:**
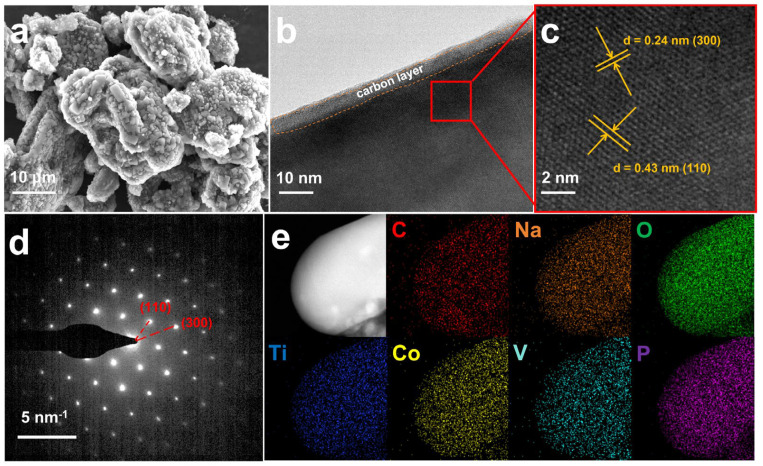
(**a**) SEM; (**b**) TEM; (**c**) high−resolution TEM; (**d**) SAED pattern; (**e**) EDS elemental mappings of NCTVP.

Scanning electron microscopy (SEM) reveals that both NCTVP and NTVP exhibit irregular particles with non-uniform sizes (Figure 2a and Figure S2). NCTVP shows a rougher surface than NTVP. Transmission electron microscopy (TEM) shows that NCTVP’s surface is covered by a carbon layer approximately 5 nm thick (Figure 2b). High-resolution TEM reveals lattice fringes with spacings of 0.43 and 0.24 nm (Figure 2c), which correspond to the (110) and (300) crystal faces of NCTVP, respectively. The selected area electron diffraction (SAED) pattern displays clear diffraction spots from the (110) and (300) planes (Figure 2d). Energy-dispersive spectroscopy (EDS) mapping exhibits the uniform dispersion of Na, Co, V, Ti, P, O, and C throughout the particles (Figure 2e).

**Figure 3:**
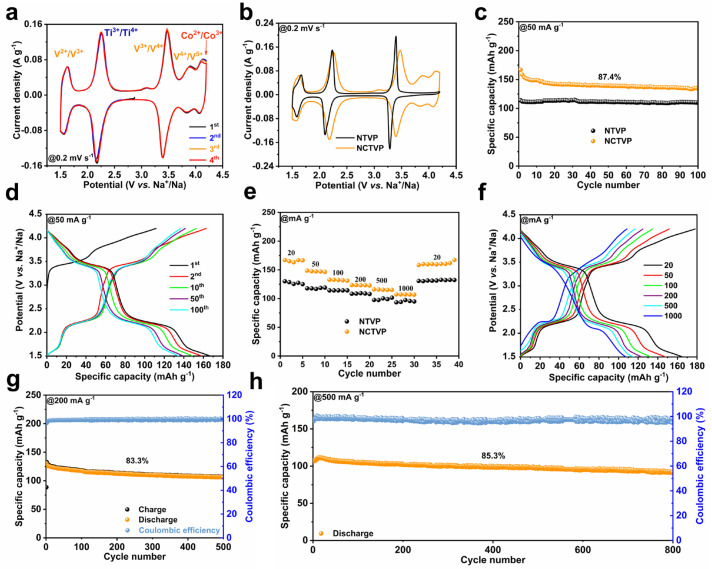
(**a**) First four CV curves of NCTVP at 0.2 mV s^−1^; (**b**) CV curves of NCTVP and NTVP at 0.2 mV s^−1^; (**c**) cycling performances of NCTVP and NTVP at 50 mA g^−1^; (**d**) GCD curves of NCTVP at 50 mA g^−1^; (**e**) rate performances of NCTVP and NTVP; (**f**) GCD curves of NCTVP at different current densities; (**g**) cycling performance of NCTVP at 200 mA g^−1^; (**h**) cycling performance of NCTVP at 500 mA g^−1^.

The sodium storage performances of NCTVP and NTVP are assessed by galvanostatic charge–discharge (GCD) and cyclic voltammetry (CV). The CV curve of NCTVP displays five pairs of redox peaks located at 4.12/4.01, 3.90/3.84, 3.46/3.40, 2.24/2.17, and 1.62/1.56 V, which are from the Co^2+^/Co^3+^, V^4+^/V^5+^, V^3+^/V^4+^, Ti^3+^/Ti^4+^, and V^2+^/V^3+^ redox couples, respectively (Figure 3a). The overlapping CV curves indicate the highly reversible intercalation/de-intercalation of Na^+^. In contrast, the CV curve of NTVP (Figure 3b and Figure S3) shows only three pairs of redox peaks from the V^3+^/V^4+^ (3.40/3.27 V), Ti^3+^/Ti^4+^ (2.23/2.12 V), and V^2+^/V^3+^ (1.67/1.56 V) couples. Compared to NTVP (Figure 3b), the CV curve of NCTVP exhibits two additional redox peaks (3.90/3.84 and 4.12/4.01 V). The appearance of the redox pair at 3.90/3.84 V suggests that Co doping activates the V^4+^/V^5+^ redox [33]. The activation of the V^4+^/V^5+^ redox reaction may result from Co-doping-facilitated electronic rearrangement in the material [34,35]. Figure 3c presents the cycling performances of NCTVP and NTVP at 50 mA g^−1^. For NCTVP, the discharge capacity reduces from 154.1 to 134.7 mAh g^−1^ in 100 cycles, achieving a retention of 87.4%. The high discharge capacity of NCTVP signifies a multi-electron redox process involving 2.8 electron transfers per formula unit. In contrast, NTVP retains a capacity of 110.8 mAh g^−1^ over 100 cycles, significantly lower than NCTVP. However, the retention of NTVP is slightly higher than NCTVP, which might be caused by the severe contraction of the crystalline structure during the oxidation of V^4+^ to V^5+^. The GCD curve (Figure 3d) of NCTVP shows a slope between 4.2 and 3.5 V from the Co^2+^/Co^3+^ and V^4+^/V^5+^ redox couples. The plateaus observed at 3.4 and 2.2 V are attributed to the V^3+^/V^4+^ and Ti^3+^/Ti^4+^ redox couples, respectively. The short slope after 2.2 V corresponds to the V^2+^/V^3+^ redox couple. In contrast, NTVP does not exhibit a discharge plateau in the 4.2–3.5 V region (Figure S4), consistent with its CV curve. Figure S5 presents the cyclability and GCD curves of NCTVP at 100 mA g^−1^. NCTVP maintains a capacity of 116.2 mAh g^−1^, with a capacity retention of 88.9% over 200 cycles. Compared to NTVP, NCTVP demonstrates superior rate performance (Figure 3e,f and Figure S6).

The capacities of NCTVP at current densities of 20–1000 mA g^−1^ are 165.1–107.0 mAh g^−1^, the maximum specific capacity approaches its theoretical value (167 mAh g^−1^), and energy density reaches 432.0 Wh kg^−1^. At any current density, the capacity of NCTVP is consistently higher than NTVP (Figure 3e,f and Figure S6). With the return of the current density to 20 mA g^−1^, the capacity recovers to 158.4 mAh g^−1^. After 500 cycles at 200 mA g^−1^ (Figure 3g) and 800 cycles at 500 mA g^−1^ (Figure 3h), the capacity retentions of NCTVP are 83.3% and 85.3%, respectively. The improvement in the electrochemical behavior of NCTVP can be attributed to Co doping. The introduction of Co provides additional V^4+^/V^5+^ and Co^2+^/Co^3+^ redox couples, which improve the capacity. However, the instability of V^4+^/V^5+^ redox may cause structural changes during charge and discharge, which have a negative impact on cycling stability [20].

**Figure 4:**
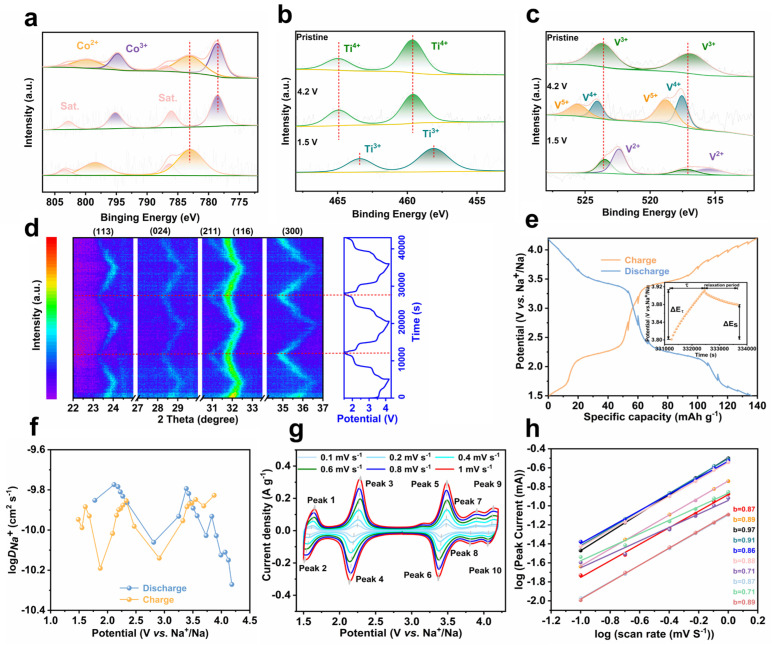
(**a**) Co 2p, (**b**) Ti 2p, and (**c**) V 2p XPS spectra; (**d**) in situ XRD patterns of NCTVP; (**e**) GITT curve; (**f**) Na^+^ diffusion coefficient of NCTVP; (**g**) CV plots of NCTVP at different scan rates; (**h**) log (peak current) as function of log (scan rate).

Ex situ X-ray photoelectron spectroscopy (XPS, Figure 4a–c) monitors the oxidation state change in transition metal elements in NCTVP during charge/discharge. The XPS survey spectrum (Figure S7) confirms the elemental composition of NCTVP. In the initial state, Co mainly exists in the form of Co^2+^ (2p_1/2_ at 802.6 eV, 2p_3/2_ at 785.4 eV), with a small portion of Co^2+^ being oxidized to Co^3+^ (2p_1/2_ at 797.7 eV, 2p_3/2_ at 781.2 eV) (Figure 4a). Upon charging to 4.2 V, Co^2+^ is oxidized to Co^3+^, and during discharge, Co^3+^ is reduced back to Co^2+^. Ti exists as Ti^4+^ (2p_1/2_ at 465.7 eV, 2p_3/2_ at 460.0 eV) in the initial state (Figure 4b). The position of Ti components does not shift during initial charging. These components shift toward lower binding energies during discharge, indicating the reduction of Ti^4+^ to Ti^3+^. As for V, it exists in the form of V^3+^ (2p_1/2_ at 523.6 eV; 2p_3/2_ at 517.0 eV) in the pristine state (Figure 4c). Upon charging to 4.2 V, four peaks appear: V^4+^ (2p_1/2_ at 524.2 eV and 2p_3/2_ at 518 eV) and V^5+^ (2p_1/2_ at 525.9 eV and 2p_3/2_ at 519.1 eV). When discharged to 1.5 V, the V peaks shift toward lower binding energies, suggesting the reduction of V^3+^ to V^2+^. The above results confirm the reversible multi-electron reaction of NCTVP during de-sodiation/sodiation.

In situ XRD discloses the crystal structure evolution of NCTVP induced by Na^+^ intercalation/de–intercalation during charging/discharging (Figure 4d). Before charging, the (113), (024), (211), (116), and (300) characteristic diffractions of NCTVP are discernable in the selected 2θ range. During charging, all the diffraction peaks shift toward higher angles continuously, demonstrating a typical solid-solution reaction mechanism. Upon discharging, the diffractions return to their original positions. The peak shifts are highly reversible during the initial several cycles, demonstrating the excellent electrochemical reversibility of NCTVP [36].

The Na^+^ diffusion coefficient ($D_{{Na}^{+}}$) of NCTVP is determined by the galvanostatic intermittent titration technique (GITT, Figure 4e). The calculated results demonstrate that the $D_{{Na}^{+}}$ value of NCTVP ranges from 10^−9^ to 10^−11^ cm^2^ s^−1^ (Figure 4f), which is comparable to that of NTVP (Figure S8) and most previously reported NASICON materials [14,15,16]. This suggests that NCTVP exhibits good Na^+^ conductivity, offering lower diffusion resistance and thereby improved performance. Electrochemical impedance spectroscopy (EIS) reveals a charge transfer resistance (R_ct_) of 70 Ω for NCTVP (Figure S9). R_ct_ is related to the capability of electron and ion transport at the electrode surface. The lower R_ct_ value of NCTVP indicates its smoother charge transfer. Efficient charge transfer is crucial for high power output, particularly during rapid charge and discharge processes.

The charge storage mechanism of NCTVP is investigated using multi-scan rate CV (Figure 4g,h). The correlation between the scan rate (*v*) and peak current (*i*) offers insights into the charge storage mechanism, as described by the following equation:

$$i=av^{b}$$

The *b*-values for peaks 1–10 are 0.87, 0.89, 0.97, 0.91, 0.86, 0.88, 0.71, 0.87, 0.71, and 0.89, respectively. When the *b*-value approaches 0.5, the charge storage mechanism is primarily diffusion-controlled. Conversely, as the *b*-value approaches 1, the mechanism is primarily capacitive, where charge is stored through rapid charge adsorption and desorption processes. These results indicate that both diffusion and capacitive mechanisms significantly contribute to the electrochemical behavior of NCTVP.

**Figure 5:**
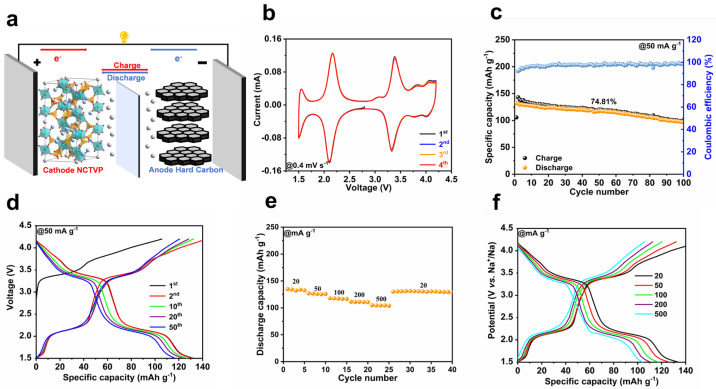
(**a**) Schematic diagram of NCTVP//HC full cell; (**b**) CV curves at 0.4 mV s^−1^; (**c**) cycling performance at 50 mA g^−1^; (**d**) GCD curves at 50 mA g^−1^; (**e**) rate performance; (**f**) GCD curves at different current densities.

Commercial hard carbon (HC) is pre-sodiated and then coupled with NCTVP for full cell assembly (Figure 5a). HC possesses a reversible capacity of ~275 mAh g^−1^ (Figure S10). The CV curves of the NCTVP//HC full cell manifests five redox pairs at 1.54/1.50, 2.18/2.09, 3.39/3.33, 3.86/3.79, and 4.10/4.02 V (Figure 5b). The shape of CV profiles remains constant as the scan rate increases (Figure S11). As presented in Figure 5c,d, the NCTVP//HC cell demonstrates an initial capacity of 133.1 mAh g^−1^ at 50 mA g^−1^. With an average discharge voltage of 2.7 V, the energy density reaches 359.3 Wh kg^−1^, exceeding that of most recently reported SIB full cells (Table S3) [37,38,39,40,41,42,43,44,45,46]. Over 100 cycles, the full cell manifests a capacity retention of 74.8%. The NCTVP//HC cell offers a capacity of 135.3 mAh g^−1^ at 20 mA g^−1^ and a capacity of over 100 mAh g^−1^ at 500 mA g^−1^ (Figure 5e,f). Obviously, the NCTVP exhibits high capacity and good cyclability in full cells, and the good full-cell performances demonstrate the potential of NCTVP for sodium storage applications.

## 4. Conclusions

In this study, we successfully constructed a NASICON-type Na_3_Co_2/3_V_2/3_Ti_2/3_(PO_4_)_3_ (NCTVP) SIB cathode material by replacing one-third of the vanadium and titanium of Na_2_VTi(PO_4_)_3_ with cobalt. Cobalt doping not only introduces the Co^3+/2+^ redox but also activates the V^5+/4+^ redox, enabling enhanced capacity and a medium discharge voltage. With multiple redox pairs, the obtained NCTVP material demonstrates a high capacity (over 160 mAh g^−1^ at 20 mA g^−1^) and energy density (432.0 Wh kg^−1^). In addition, NCTVP also demonstrates good cyclability (87.4% retention over 100 cycles at 50 mA g^−1^). The NCTVP//HC full cell also manifests good cycling performance and high energy density, showing significant potential for practical applications.

## Data Availability

The data presented in this study are available upon request from the corresponding author. The data are not publicly available due to privacy restrictions.

## Supplementary Materials

The following supporting information can be downloaded at https://www.mdpi.com/article/10.3390/ma18112419/s1, Table S1: Detailed structural information of NCTVP derived from Rietveld Refinement; Table S2: Detailed structural information of NTVP derived from Rietveld Refinement; Table S3: Comparison of the electrochemical performances of NCTVP with previously reported phosphate cathodes; Figure S1: Rietveld refinement of the XRD pattern of NTVP (a) and XRD pattern of NCTVP (b); Figure S2: SEM images of NTVP; Figure S3: CV curves at different scan rates from 0.1 to 1.0 mV s^−1^ of NTVP (a) and first four cycles at a scan rate of 0.2 mV s^−1^ (b); Figure S4: The GCD curves of NTVP at 50 mA g^−1^; Figure S5: The cycling performance of NCTVP at 100 mA g^−1^ (a) and the GCD curves of NCTVP at 100 mA g^−1^ (b); Figure S6: The GCD curves of NTVP at different current densities; Figure S7: XPS survey spectrum of the NCTVP; Figure S8: GITT profile (a) and Na^+^ diffusion coefficients of NTVP (b) and potentials for NCTVP and NTVP as a function of the degree of sodiation (c); Figure S9: EIS plots of the NCTVP and NTVP; Figure S10: GCD curves of HC at 50 mA g^−1^; Figure S11: CV curves of NCTVP//HC full cell at different scan rates. Refs. [33,36,37,38,39,40,41,42,43,45,46] are also cited in Supplementary Materials.
