# Supplementary material for: Cobalt Doping of Na2VTi(PO4)3 Enables a High-Energy NASICON-Type Cathode Material for Sodium-Ion Batteries
Source: Materials (Basel). 2025 May 22;18(11):2419. doi: 10.3390/ma18112419 (PMC12156873; doi:10.3390/ma18112419)
Supplement: Supplementary file 1 [file materials-18-02419-s001.zip › materials-3610061-supplementary.pdf]

# Supporting Information

# Cobalt-doping of $Na_2VTi(PO_4)_3$ enables a high-energy NASICON-type cathode material for sodium-ion batteries

Yu Zhang [1], Mengyao Wang [1], Hao Fan [1], Chenyang Huang [1], Mingfei Liu [1], Xiaofa Liang [1], Ping Hu [2, 3]*, Xuanpeng Wang [3], Qin Wang [4], Fei Lv [4], Liang Zhou [1, 5]*

[1] State Key Laboratory of Advanced Technology for Materials Synthesis and Processing, Wuhan University of Technology, Wuhan, 430070, P. R. China
[2] Key Laboratory of Intelligent Sensing System and Security of the Ministry of Education, Hubei Key Laboratory of Micro-Nanoelectronic Materials and Devices, School of Microelectronics, Hubei University, Wuhan 430062, P. R. China
[3] Zhongyu Feima New Material Technology Innovation Center (Zhengzhou) Co. Ltd., High Technology Industrial Development Zone, No. 60 Xuelan Road, Zhengzhou, 450001, P. R. China
[4] Hubei Longzhong Laboratory, Wuhan University of Technology (Xiangyang Demonstration Zone), Xiangyang, 441000, Hubei, P. R. China
[5] Hubei Wanrun New Energy Technology Co., Ltd., No.557, Tianma Road, Yunyang Economic Development Zone, Shiyan 442003, P. R. China
* Correspondence: liangzhou@whut.edu.cn (L.Z.); huping316@hubu.edu.cn (P.H.)

**Characterization.** X-ray diffraction (XRD) measurements are performed on a Bruker D8 Discover equipment utilizing the X-ray diffractometer with Cu Kα X-ray source (λ = 1.5406 Å). Scanning electron microscope (SEM) images are collected using a JEOL-7100F microscope. Transmission electron microscopy (TEM) images and energy dispersive spectroscopy (EDS) mappings are collected on a JEOL JEM - 2100F STEM/EDS microscope. Raman spectroscopy is performed with the Horiba Lab RAM HREvolution. The chemical bonding information of the materials is obtained by Fourier transform infrared spectroscopy (FT-IR, Nicolet iS50). X-ray photoelectron spectroscopy (XPS) spectra are collected with a VG Multilab 2000. An ASAP 2020 analyzer is used to acquire nitrogen adsorption-desorption isotherms to assess the BET-specific surface area and pore size distribution of the materials. Thermogravimetric analysis (TGA) tests are performed using a type STA-449C thermogravimetric analyzer in air with a ramp rate of 10 °C $min^{-1}$.

**Electrochemical Characterization.** The electrochemical properties were tested with CR2016 coin-type cells. The cathode was fabricated by pressing a slurry containing 70 mg active material, 20 mg acetylene black, and 10 mg polyvinylidene fluoride which was dissolved in N-methyl-2-pyrrolidone, and placed onto an aluminum foil and dried under vacuum at 60 °C for 12 h. The areal mass loading of active material was approximately 1 – 1.2 mg $cm^{-2}$. Sodium disks were employed as the counter and reference electrodes; 1 M $NaPF_6$ in diethyl carbonate (DEC) and ethylene carbonate (EC) (1:1 by weight) with 0.2 wt.% $NaClO_4$ and 1 wt.% tris(trimethylsilyl) phosphate (TMSP) was used as the electrolyte; Whatman glass microfiber paper was used as the

separator. Galvanostatic charge/discharge tests were performed in the potential window of 1.5 – 4.2 V *vs*. Na/$Na^+$ using a multichannel battery testing system (LAND CT2001A). Cyclic voltammetry (CV) and Electrochemical Impedance Spectroscopy (EIS) was performed using CHI760E. EIS tests at frequencies ranging from 0.1 Hz to 100 kHz are performed using the Autolab PGSTAT 302N electrochemical workstation.

The full cells are assembled with NCTVP as the cathode, pre-sodiated hard carbon (HC) as the anode, Whatman GF/D glassfibre diaphragm as the separator, and the electrolyte was identical to that used in half-cell testing. The HC anodes are prepared by dispersing HC (70 wt.%), acetylene black (20 wt.%), and polyvinylidene fluoride (10 wt.%) in N-methyl pyrrolidone, and then coated onto a copper foil and vacuum-dried at 70 °C for 10 h. The HC electrodes are 12 mm diameter discs with area load in the range of 0.5-0.7 mg $cm^{-2}$. For the pre-sodiation of HC, CR2016 cells are assembled using HC as the work electrode, Na as the counter electrode, Whatman GF/D glassfibre diaphragm as the separator, and 1.0 M $NaPF_6$ in tetrahydrofuran as the electrolyte. After four cycles at a current density of 50 mA $g^{-1}$ in a potential window of 0.01-2.0 V, the HC electrodes are disassembled from the coin cell for full cell assembly.

*In-situ* XRD experiment was performed on a D8 Discover X-ray diffractometer equipped with a planar detector. The cathode was prepared with the ratio of 6 (active material) : 3 (acetylene black): 1 (polytetrafluoroethylene). Then, the tableted electrode slice was cut into square slices with an area of ~0.785 $cm^2$ and a thickness of ~0.2 mm. The prepared cathode was placed on the backside of the Be window (X-ray transparent), which also served as the current collector. The in-situ XRD signals were collected in

the 2θ range of $20^{o}$ to $37^{o}$ with a still mode during the initial charge/discharge process.

The energy density of the electrode was calculated using the standard equation:

$$E = C * V$$

where $E$ is the energy density (Wh $kg^{-1}$), $C$ is the specific capacity (mAh $g^{-1}$), $V$ is the medium discharge voltage (V).

**Table S1.** Detailed structural information of NCTVP derived from Rietveld Refinement.

| Space group = *R-3c* | | $R_p$ = 4.35 % | $R_{wp}$ = 6.00 % | |
|---|---|---|---|---|
| *a* (Å) = 8.7016 | | *c* (Å) = 21.7675 | *a/b* = 1.0000 | |
| *b/c* = 0.40 | | *c/a* = 2.50 | $V(Å^3)$ = 1427.3770 | |
| Atom | Wyckoff site | *X* | *Y* | *Z* |
| Co1 | 12c | 0.000000 | 0.000000 | 0.142865 |
| P1 | 18e | 0.266753 | 0.000000 | 0.250000 |
| O1 | 36f | -0.012462 | 0.043069 | 0.161658 |
| O2 | 36f | -0.012462 | 0.202041 | 0.087454 |
| Na1 | 6b | 0.000000 | 0.000000 | 0.000000 |
| Na2 | 18e | 0.350434 | 0.000000 | 0.250000 |
| Ti1 | 12c | 0.000000 | 0.000000 | 0.142865 |
| V1 | 12c | 0.000000 | 0.000000 | 0.142865 |

**Table S2.** Detailed structural information of NTVP derived from Rietveld Refinement.

| Space group = *R-3c* | | $R_p$ = 4.63 % | $R_{wp}$ = 6.50 % | |
|---|---|---|---|---|
| *a* (Å) = 8.6148 | | *c* (Å) = 21.8465 | *a/b* = 1.0000 | |
| *b/c* = 0.39 | | *c/a* = 2.53 | $V(Å^3)$ = 1404.1320 | |
| Atom | Wyckoff site | *X* | *Y* | *Z* |
| Ti1 | 12c | 0.000000 | 0.000000 | 0.145210 |
| P1 | 18e | 0.289764 | 0.000000 | 0.250000 |
| O1 | 36f | 0.021545 | 0.204072 | 0.199813 |
| O2 | 36f | 0.186356 | 0.166313 | 0.088018 |
| Na1 | 6b | 0.000000 | 0.000000 | 0.000000 |
| Na2 | 18e | 0.608190 | 0.000000 | 0.250000 |
| V1 | 12c | 0.000000 | 0.000000 | 0.145210 |

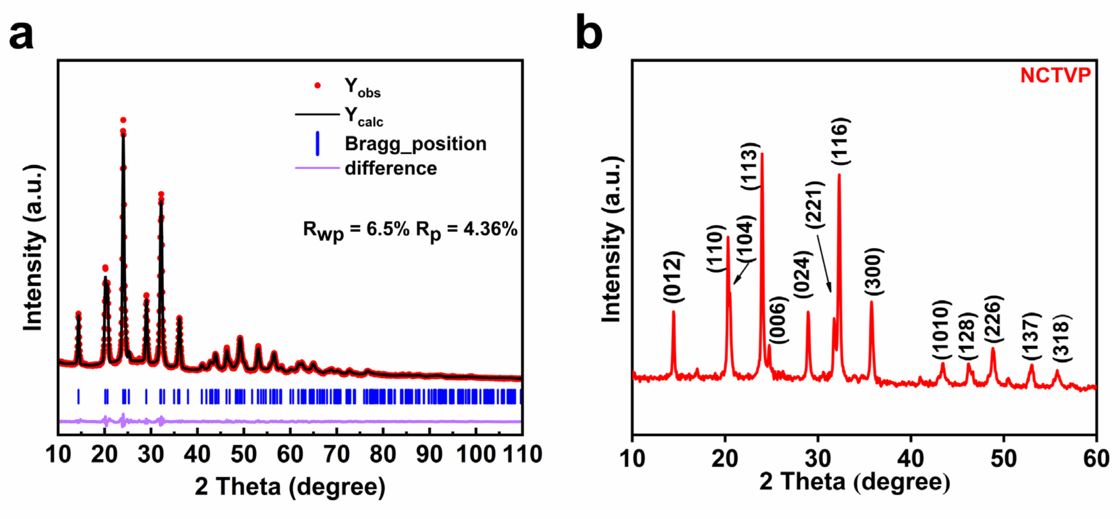


**Figure S1**. Rietveld refinement of the XRD pattern of NTVP (a) and XRD pattern of NCTVP (b).

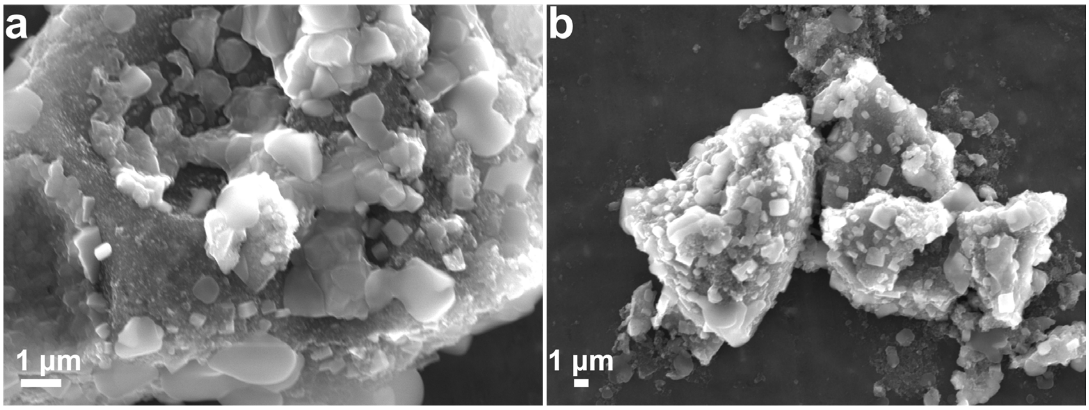


**Figure S 2.** SEM images of NTVP.

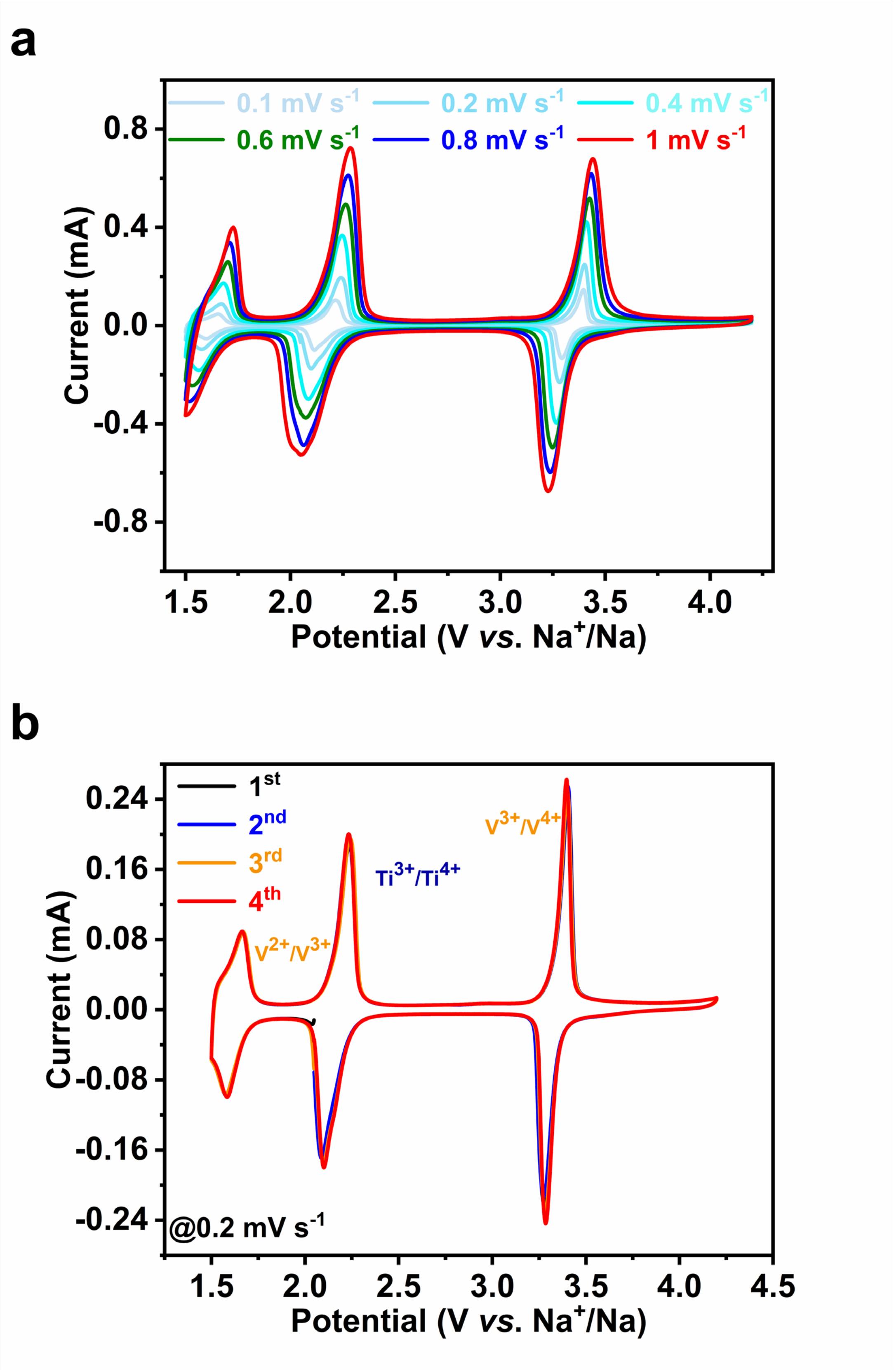


**Figure S3.** CV curves at different scan rates from 0.1 to 1.0 mV $s^{-1}$of NTVP (a) and first four cycles at a scan rate of 0.2 mV $s^{-1}$ (b).

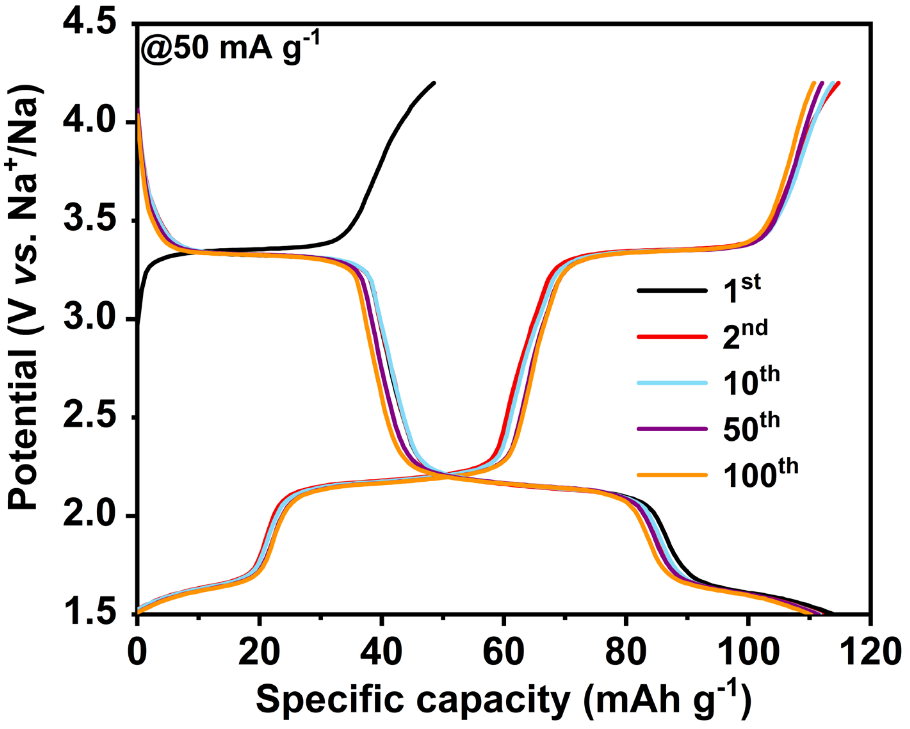


**Figure S4.** The GCD curves of NTVP at 50 mA $g^{-1}$.

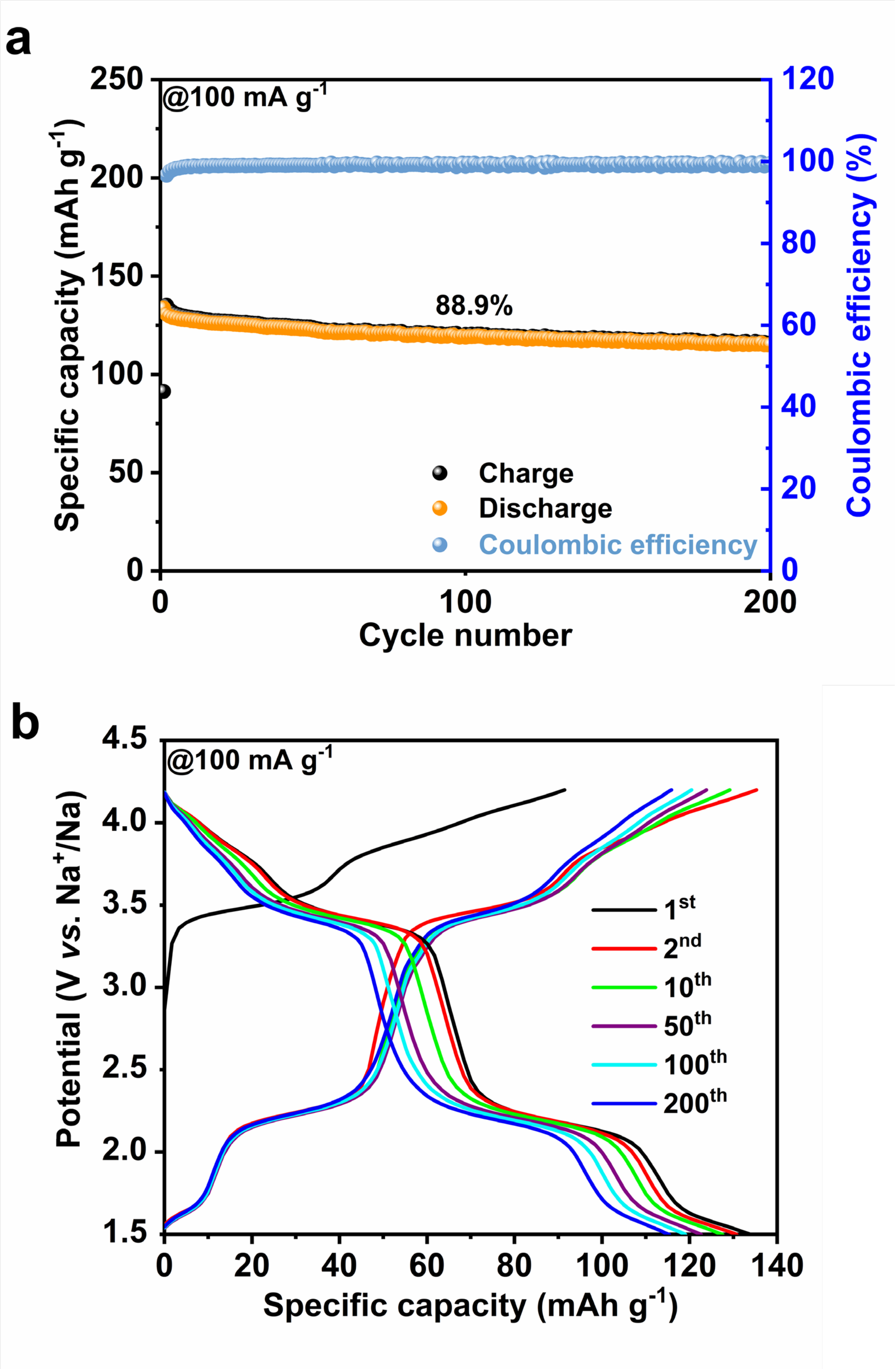


**Figure S5.** The cycling performance of NCTVP at 100 mA $g^{-1}$ (a) and the GCD curves of NCTVP at 100 mA $g^{-1}$ (b).

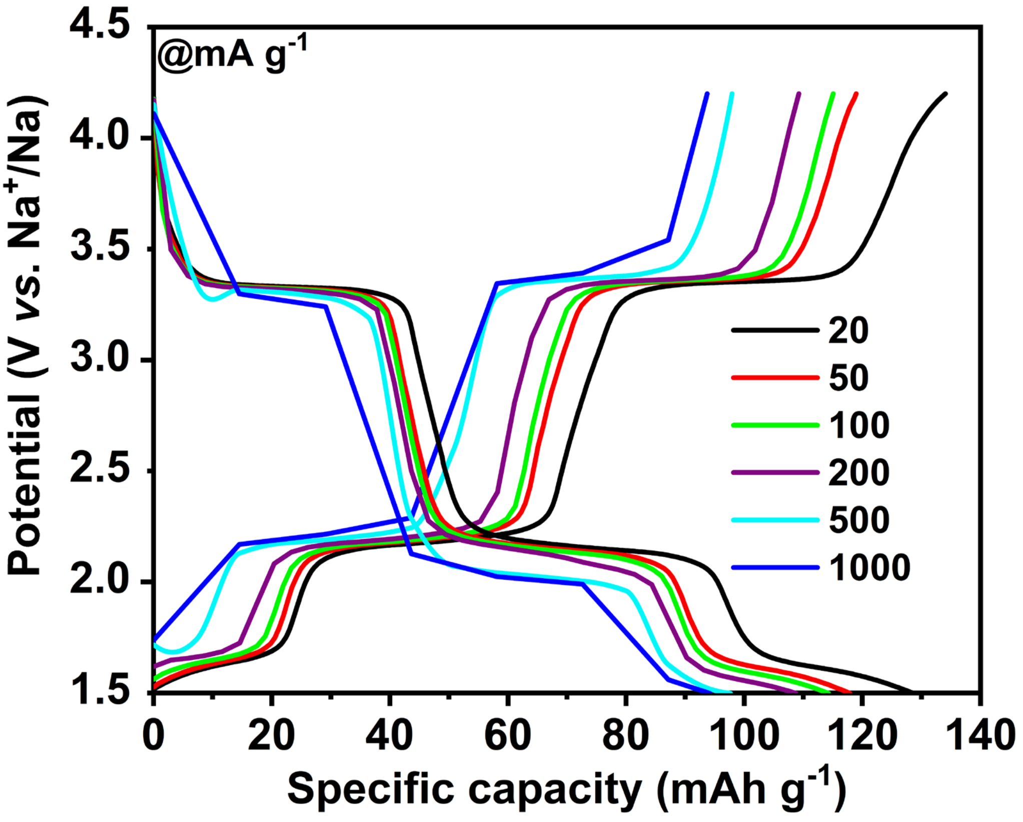


**Figure S6.** The GCD curves of NTVP at different current densities.

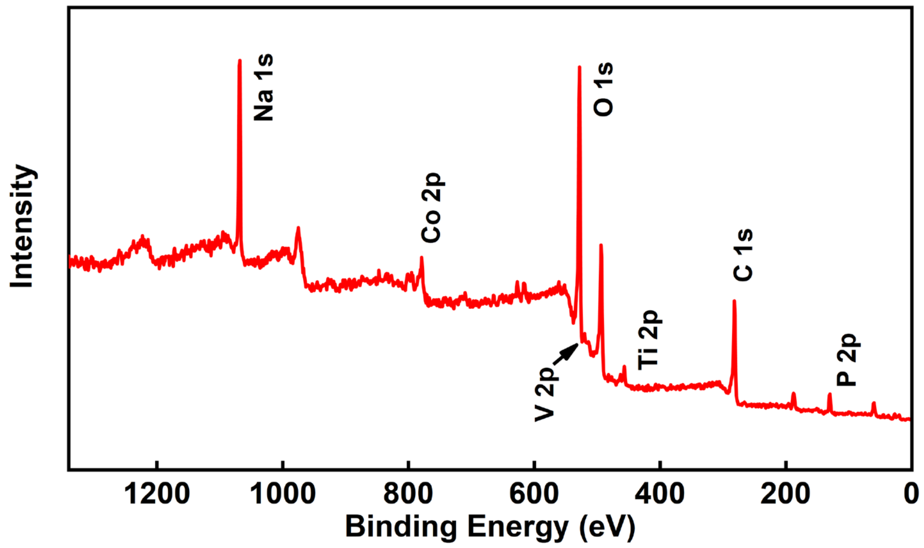


**Figure S7.** XPS survey spectrum of the NCTVP.

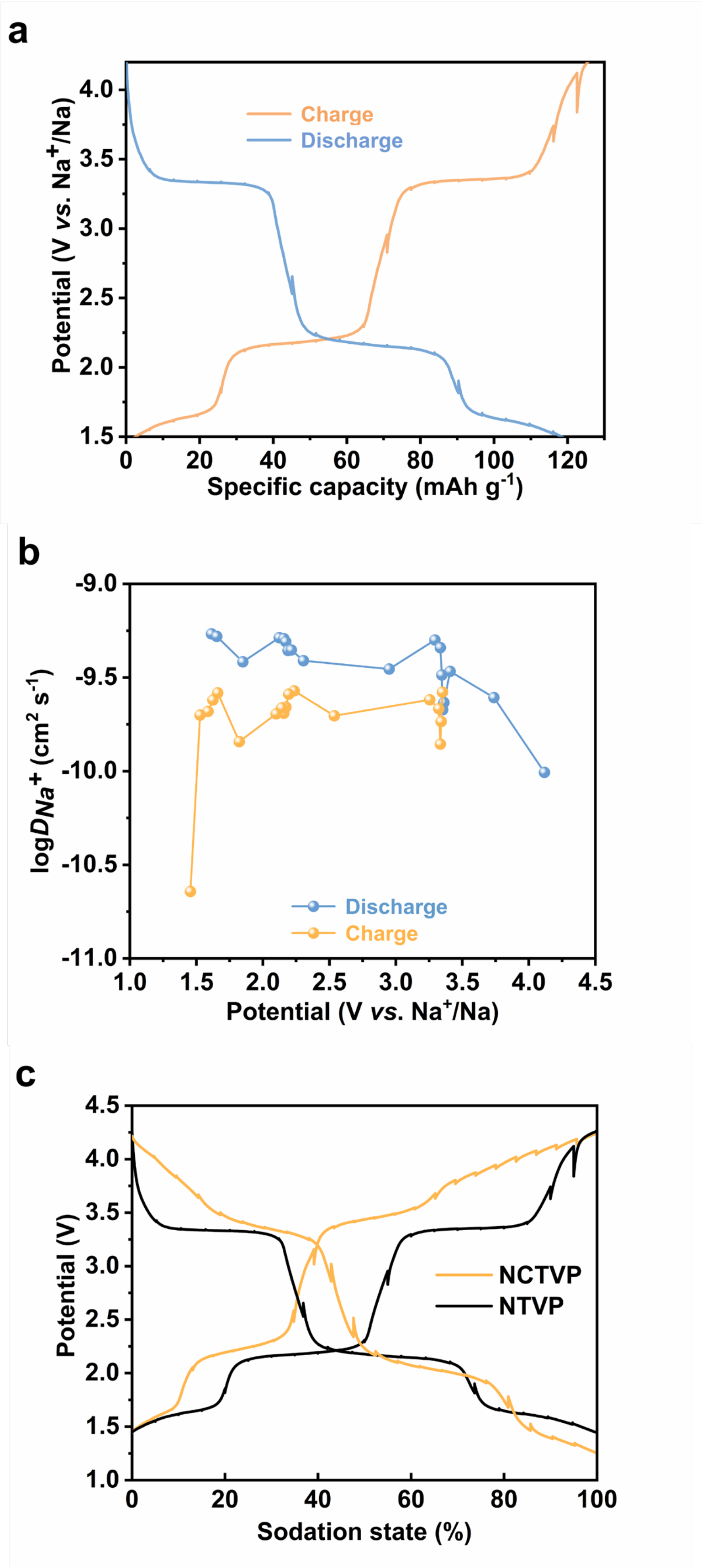


**Figure S8.** GITT profile (a) and $Na^{+}$ diffusion coefficients of NTVP (b) and potentials for NCTVP and NTVP as a function of the degree of sodiation (c).

The $D_{Na+}$ values can be calculated based on the following equation:

$$D_{Na^+} = \frac{4}{\pi\tau}\left(\frac{m_B V_M}{M_B S}\right)^2 \left(\frac{\Delta E_S}{\Delta E_\tau}\right)^2 \qquad \left(\tau \ll \frac{L^2}{D_{Na^+}}\right)$$

where $\tau$ is the relaxation time, $S$ is the electrode/electrolyte contact area, $m_B$, $M_B$ and $V_M$ are the active material mass, molar mass, and molar volume of the electrode, respectively, $L$ is the average radius of the material particles, $\Delta E_\tau$ is the variation of the voltage during the constant current pulse, and $\Delta Es$ is the difference in the voltage during the open-circuit period.

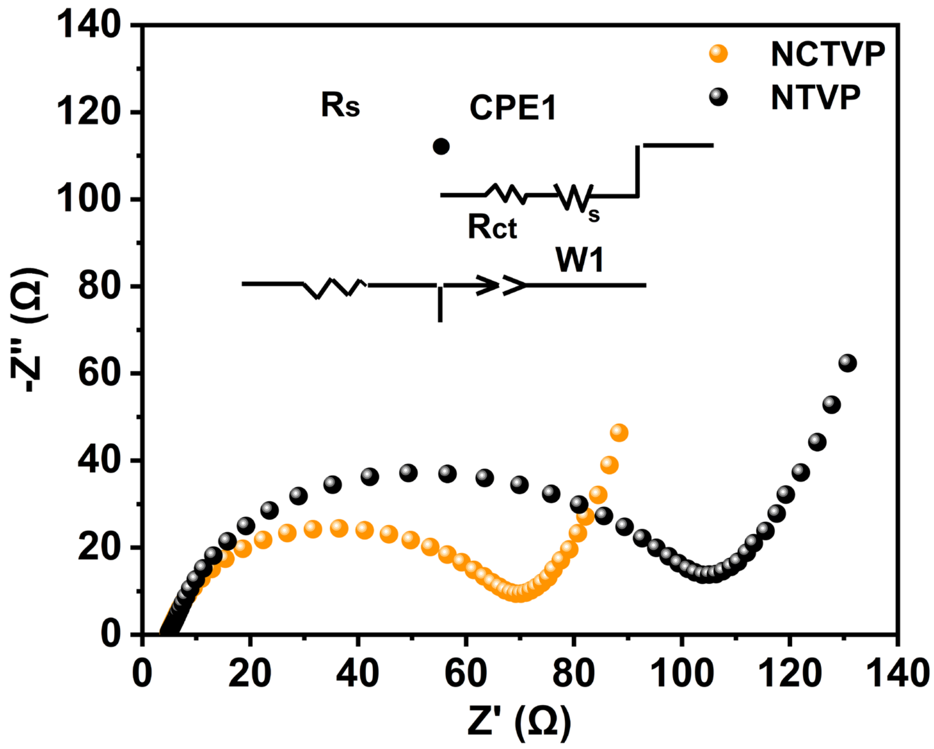


**Figure S9.** EIS plots of the NCTVP and NTVP.

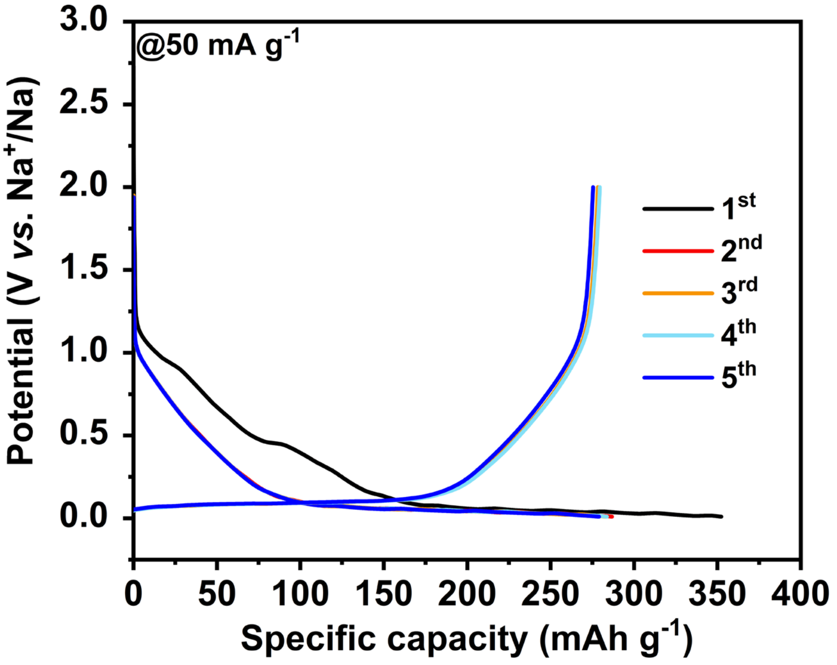


**Figure S10.** GCD curves of HC at 50 mA $g^{-1}$.

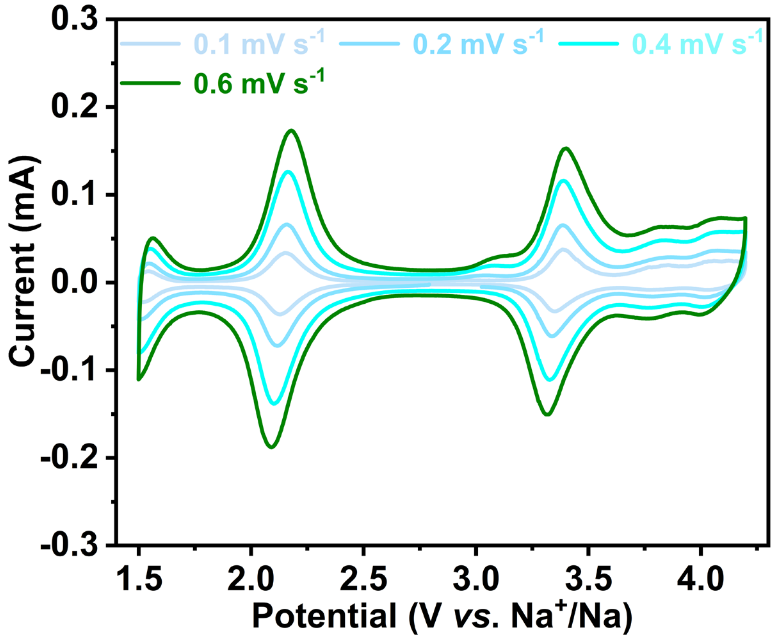


**Figure S11.** CV curves of NCTVP // HC full cell at different scan rates.

**Table S3.** Comparison of the electrochemical performances of NCTVP with previously reported phosphate cathodes.

| Materials | Discharge capacity | Energy density | Ref |
|---|---|---|---|
| $Na_3MnTi(PO_4)_3$//HC | 91.0 mAh $g^{-1}$ at 0.2C | 301 Wh $kg^{-1}$ | [1] |
| $Na_3V_{1.5}Al_{0.3}Fe_{0.2}(PO_4)_3$//HC | 116.2 mAh $g^{-1}$ at 0.1 C | 254 Wh $kg^{-1}$ | [2] |
| $Na_{3.34}Mn_{1.2}Ti_{0.8}(PO_{3.98}F_{0.02})_3$//CC | 80.4 mAh $g^{-1}$ at 0.1 C | 246 Wh $kg^{-1}$ | [3] |
| $Na_{3.7}VAl_{0.3}Mn0_{.7}(PO_4)_3$//HC | 110.4 mAh $g^{-1}$ at 0.5 C | 344 Wh $kg^{-1}$ | [4] |
| $Na_4Fe_{2.91}Cd_{0.09}(PO_4)_2P_2O_7$//HC | 97.1 mAh $g^{-1}$ at 13 mA $g^{-1}$ | 180 Wh $kg^{-1}$ | [5] |
| $Na_{2.8}Mn_{0.4}V_{1.0}Ti_{0.6}(PO_4)_3$//HC | 122.4 mAh $g^{-1}$ at 0.1 C | 266Wh $kg^{-1}$ | [6] |
| $Na_4VFe(PO_4)_3$//HC | 121.5 mAh $g^{-1}$ at 0.1 C | 306 Wh $kg^{-1}$ | [7] |
| $Na_3MnHf(PO_4)_3$//HC | 82.5 mAh $g^{-1}$ at 0.1C | 225 Wh $kg^{-1}$ | [8] |
| $Na_4MnV(PO_4)_3$/NC @CNTs//HC | 118.2 mAh $g^{-1}$ at 0.1 C | 325 Wh $kg^{-1}$ | [9] |
| $Na_3V_{1.8}(CrMnFeZnAl)_{0.2}(PO_4)_3$//HC | 112.2 mAh $g^{-1}$ at 0.5 C | 201 Wh $kg^{-1}$ | [10] |
| $Na_{3.1}MnTi_{0.9}V_{0.1}(PO_4)_3$//HC | 93.5 mAh $g^{-1}$ at 0.1 C | 320 Wh $kg^{-1}$ | [11] |
| **$Na_3Co_{2/3}Ti_{2/3}V_{2/3}(PO_4)_3$//HC** | **133.1 mAh $g^{-1}$ at 50 mA $g^{-1}$** | **359Wh $kg^{-1}$** | **This work** |

References

[1] Zhu T, Hu P, Cai C, et al. Dual carbon decorated $Na_3MnTi(PO_4)_3$: a high-energy-density cathode material for sodium-ion batteries[J]. Nano Energy, 2020, 70: 104548.

[2] Li S Y, Yin Q M, Gu Z Y, et al. Cation/Anion-Dual regulation in $Na_3MnTi(PO_4)_3$ cathode achieves the enhanced electrochemical properties of Sodium-Ion batteries[J]. Journal of Colloid and Interface Science, 2024, 664: 381-388.

[3] Liu X, Zhu C, Xu T, et al. Multifunctional-Element doping of NASICON-Structured cathode enables High-Rate and stable sodium storage[J]. Chemical Engineering Journal, 2024, 497: 154304.

[4] Park J Y, Shim Y, Kim Y I, et al. An iron-doped NASICON type sodium ion battery cathode for enhanced sodium storage performance and its full cell applications[J]. Journal of Materials Chemistry A, 2020, 8(39): 20436-20445.

[5] Shen X, Su Y, He S, et al. A zero-strain Na-deficient NASICON-type $Na_{2.8}Mn_{0.4}V_{1.0}Ti_{0.6}(PO_4)_3$ cathode for wide-temperature rechargeable Na-ion batteries[J]. Journal of Materials Chemistry A, 2023, 11(31): 16860-16870.

[6] Sun C, Ni Q, Li M, et al. Improving rate performance by inhibiting Jahn-Teller effect in Mn-based phosphate cathode for Na-Ion batteries[J]. Advanced Functional Materials, 2024, 34(7): 2310248.

[7] Wu F, Ma H, Ye X, et al. Structural modulation of $Na_4Fe_3(PO_4)_2P_2O_7$ via cation engineering towards high-rate and long-cycling sodium-ion batteries[J]. Journal of Colloid and Interface Science, 2025, 679: 132-140.

[8] Zhang X, Jiang L, Xu C, et al. Designing high-performance phosphate cathode toward Ah-level Na-ion batteries[J]. Energy Storage Materials, 2024, 72: 103764.

[9] Zhu Y, Xu H, Bao Y, et al. Investigating the effect of calcination temperature on the electrochemical properties of $Na_4MnV(PO4)_3$/NC@ CNTs cathode materials for sodium ion batteries[J]. Journal of Energy Storage, 2024, 90: 111910.

[10] Zhou Y, Xu G, Lin J, et al. A multicationic-substituted configurational entropy-enabled NASICON cathode for high-power sodium-ion batteries[J]. Nano Energy, 2024, 128: 109812.

[11] Hu P, Cai C, Li X, et al. V doping in NASICON-structured $Na_3MnTi(PO_4)_3$ enables high-energy and stable sodium storage[J]. Advanced Functional Materials, 2024, 34(5): 2302045.
